# Impact of Reactive Oxygen and Nitrogen Species Produced by Plasma on Mdm2–p53 Complex

**DOI:** 10.3390/ijms22179585

**Published:** 2021-09-03

**Authors:** Pankaj Attri, Hirofumi Kurita, Kazunori Koga, Masaharu Shiratani

**Affiliations:** 1 Center of Plasma Nano-Interface Engineering, Kyushu University, Fukuoka 819-0395, Japan; siratani@ed.kyushu-u.ac.jp; 2 Graduate School of Information Science and Electrical Engineering, Kyushu University, Fukuoka 819-0395, Japan; 3 Department of Applied Chemistry and Life Science, Toyohashi University of Technology, Toyohashi 441-8580, Aichi, Japan; kurita@chem.tut.ac.jp; 4 Faculty of Information Science and Electrical Engineering, Kyushu University, Fukuoka 819-0395, Japan; koga@ed.kyushu-u.ac.jp; 5 Center for Novel Science Initiatives, National Institute of Natural Science, Tokyo 105-0001, Japan

**Keywords:** Mdm2–p53, plasma treatment, molecular dynamic (MD) simulations

## Abstract

The study of protein–protein interactions is of great interest. Several early studies focused on the murine double minute 2 (Mdm2)–tumor suppressor protein p53 interactions. However, the effect of plasma treatment on Mdm2 and p53 is still absent from the literature. This study investigated the structural changes in Mdm2, p53, and the Mdm2–p53 complex before and after possible plasma oxidation through molecular dynamic (MD) simulations. MD calculation revealed that the oxidized Mdm2 bounded or unbounded showed high flexibility that might increase the availability of tumor suppressor protein p53 in plasma-treated cells. This study provides insight into Mdm2 and p53 for a better understanding of plasma oncology.

## 1. Introduction

Overexpression of murine double minute 2 (Mdm2), detected in many malignancies such as lung cancer, liver cancer, breast cancer, colorectal cancer, esophagogastric cancer, etc., has resulted in the inactivation of tumor suppressor protein p53 [1,2]. p53 plays an essential role in inhibiting the malignant transformation by controlling the cell cycle, apoptosis, and DNA repair [3,4]. The disruption of the Mdm2–p53 complex is a target for developing many therapeutic agents [5]. The chemical agents were synthesized to inhibit the inactivation of p53 by Mdm2 to enhance the anticancer activity [6,7]. Numerous peptides that mimic p53 have been synthesized to use as inhibitors, but peptides’ poor membrane permeability limited their use in cancer treatment [8,9,10]. Very recently, Sahin et al. reported that cell lines with high expression of Mdm2 showed resistance to T-cell-mediated tumor killing [11]. It was reported that Mdm2 overexpression could result in therapeutic resistance to radiotherapy, chemotherapy, etc. [1]. However, the role of the Mdm2–p53 complex is missing in plasma oncology.

Plasma consists of ionized gas that contains a mixture of positively charged ions, electrons, and neutral particles. The discharge under atmospheric conditions is also commonly known as non-thermal atmospheric pressure plasma (NTAPP) or cold atmospheric plasma (CAP). Plasma generates plenty of reactive chemical species, both short-lived and long-lived, such as reactive oxygen and nitrogen species (RONS). In the last few years, CAP use in cancer treatment has increased tremendously [12,13,14,15,16]. Adil et al. showed 58.07, 61.7, and 68% of cancer cells death for AMJ13 (human breast cancer), MCF7 (human breast cancer), and AMN3 (mouse mammary adenocarcinoma) cell lines, respectively, while there was an insignificant cytotoxic effect on the HBL (normal breast tissue) cell line after CAP treatment [12]. Irani et al. showed the anticancer effect of CAP and gold nanoparticle combination on HCT-116 cells (human colorectal cancer cells) [13]. Vaquero et al. showed that CAP reduced the CCA (cholangiocarcinoma) progression through DNA damage [14]. The apoptotic cell death of A549 lung cancer cells within a 3D collagen matrix, was shown by Karki et al. [15]. Additionally, direct CAP and CAP-treated liquid was used to kill the chemoresistant cell lines (temozolomide (TMZ)-resistant glioblastoma cells, tamoxifen sensitivity breast cancer cells, tumor-necrosis-factor-related apoptosis-inducing ligand (TRAIL)-resistant colorectal cancer cells, and 5-fluorouracil-resistant hepatocarcinoma cells, etc.) [16,17,18,19,20,21]. Besides the reported achievements of CAP to treat cancer, CAP’s action is still not known entirely. Bauer noted that extracellular RONS could trigger membrane-associated intracellular signaling, which resulted in cell death through the deactivation of the catalase enzyme by singlet oxygen (^1^O_2_) [22]. We recently reported that CAP treatment on cancer cells decreases NOX1 expression in A375 melanoma cells and oxidizes the NADPH oxidase activator (Noxa 1) SH3 protein [23]. Decreased NOX1 expression might result in low production of O_2_^●−^, which affects the generation of ^1^O_2_. Hence, catalase enzymes remain active and decompose the excess H_2_O_2_ in cancer cells, so there will be an alternative pathway for CAP-induced cancer cell death. To do so, we investigated the structural changes in Mdm2, p53, and Mdm2–p53 complex before and after possible plasma oxidation through molecular dynamic (MD) simulation. This study provides insight into Mdm2 and p53 that will help in further understanding plasma oncology.

## 2. Results

The previously reported experimental and computational studies showed the effect of CAP-induced oxidation on various proteins structure such as Noxa1SH3 [23], SARS-CoV-2-CTD spike protein [24], bacteriorhodopsin [25], horseradish peroxidase [26], MTH1880 [27], lysozyme [28,29], myoglobin [30,31], lipase [32], hemoglobin [33], α-chymotrypsin [34], and lactate dehydrogenase (LDH) enzyme [35], that results in the structural distortion of proteins. Based on the possible oxidation products of amino acids after plasma treatment, we modified the selective amino acids of Mdm2 and p53, which play an essential role in the Mdm2–p53 complex, and performed simulations. As reported earlier, the p53-binding region of Mdm2 consists of a hydrophobic groove with 70% of the atoms at the nonpolar interface [6,7]. Recently, Zou et al. showed that binding of intrinsically disordered protein p53 prefers the induced-fit mechanism that proceeds stepwise [36]. Authors noticed that unbinding of p53 firstly unfolds and then unbinds from Mdm2 protein [36]. In another study, 182 independent continuous binding pathways were obtained for Mdm2 and intrinsically disordered p53 [37]. This shows Mdm2–p53 binding is of great interest.

**Figure 1:**
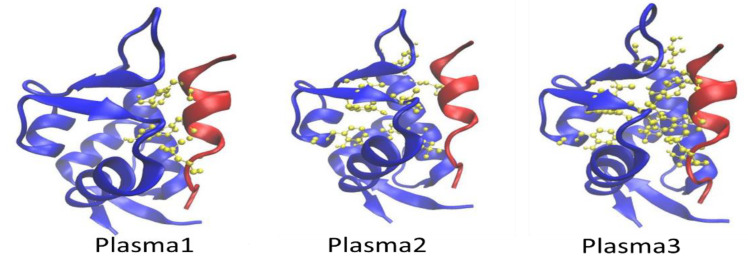
Schematic representation of the different types of oxidized form of Mdm2–p53 proteins plasma1, plasma2, and plasma3. The Mdm2–p53 protein PDB IDs is 1YCR.

p53 hydrophobic amino acids Phe19, Trp23, and Leu26 are located face-to-face on the helix’s same side towards the Mdm2 protein [6]. p53 is surrounded by hydrophobic residues of Mdm2 such as Leu54, Leu57, Ile61, Met62, Tyr67, Val75, Val93, Phe86, Ile99, Phe91, and Ile103 [6]. Hence, we modified the hydrophobic amino acids of Mdm2 and p53 based on the reported amino acid product after plasma oxidation [38] and divided it into three types. In type 1, we used the oxidized plasma product of Phe19, Trp23, and Leu26 of p53, amino acids called plasma1; see Figure 1. For type 2, we oxidized only Mdm2 amino acids located in a hydrophobic groove such as Leu54, Leu57, Met62, Tyr67, Val75, Val93, Phe86, and Phe91, called plasma2, and modified amino acids of both p53 and Mdm2, known as plasma3 (type 3 form) (Figure 1). The GROMACS force field parameters [39,40] for Ile plasma oxidation products are not available, so we have not included Ile61, Ile99, and Ile103 amino acids modification in Mdm2 for plasma2 and plasma3.

### 2.1. MD Simulation of p53 and Mdm2 before and after Possible CAP Modification

**Figure 2:**
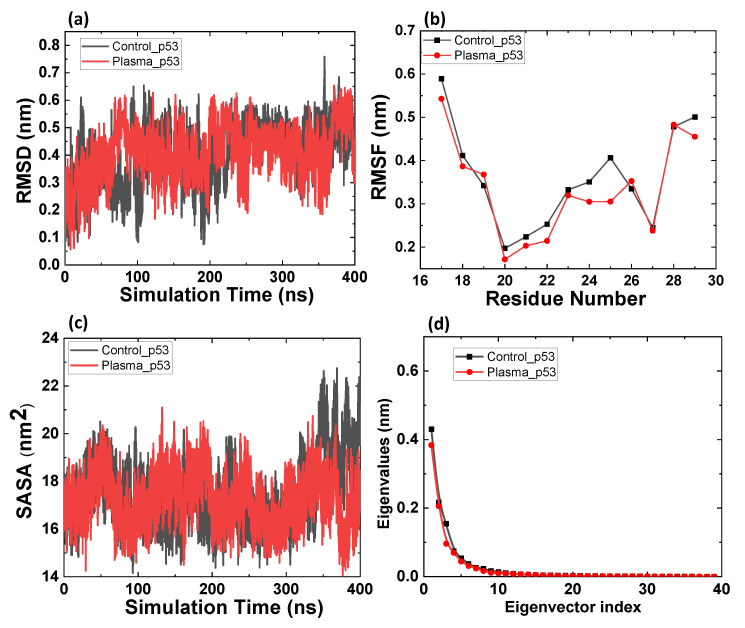
MD simulation results of the native and oxidized p53 protein. (**a**) Root mean square deviation (RMSD) of the C_α_ atoms, (**b**) root mean square fluctuations (RMSFs), (**c**) solvent-accessible surface area (SASA), and (**d**) comparison of the eigenvalues plotted against the corresponding eigenvector indices obtained from the C_α_ covariance matrix.

We used MD simulation to calculate the root-mean-square deviation (RMSD), root mean square fluctuation (RMSF), solvent-accessible surface area (SASA), and principal component analysis (PCA) for native and oxidized p53 and Mdm2 proteins, to evaluate the CAP-induced oxidation effect on stability and flexibility of the proteins. We modified Gln, Leu, Met, Phe, Trp, Tyr, and Val to 4-hydroxyglutamine, 4-hydroxyleucine, methionine sulfoxide, 2,3-dihydroxyphenylalanine, 6-hydroxytryptophan, 3,4-dihydroxyphenylalanine, and 3-hydroxyvaline. Figure 2a shows the change in RMSD values of p53 before and after CAP-induced modification. The average RMSD values were 0.50 ± 0.06 and 0.50 ± 0.10 nm for control and oxidized p53, respectively, from the last 50 ns simulation. No change in RMSD value before and after p53 oxidation reveals the stability p53 against possible plasma oxidation. RMSF results in Figure 2b also indicated the slightly reduced fluctuations for oxidized p53. Figure 2c shows the changes in SASA of p53 with and without oxidized amino acids. The average SASA was 19 ± 1 and 17 ± 1 nm^2^, for control and modified p53, respectively. The results indicate that the surface area accessible to the water decreases after modification of amino acids (Phe19, Trp23, and Leu26). Further, we calculated the properties of the motions characterized by the principal components. The magnitudes of p53 control and oxidized eigenvalues are similar, as seen in Figure 2d; these results are correlated with RMSD analysis.

**Figure 3:**
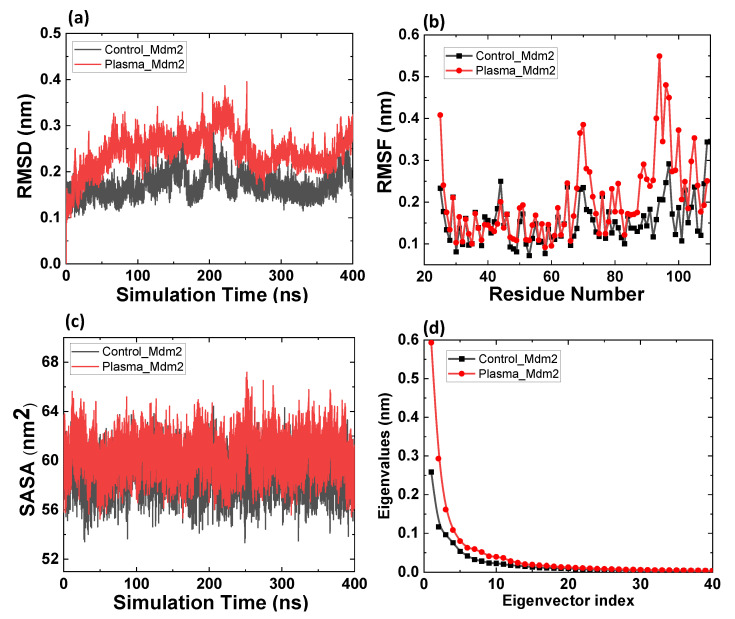
MD simulation results of the native and oxidized Mdm2 protein. (**a**) Root mean square deviation (RMSD) of the C_α_ atoms, (**b**) root mean square fluctuations (RMSFs), (**c**) solvent-accessible surface area (SASA), and (**d**) comparison of the eigenvalues plotted against the corresponding eigenvector indices obtained from the C_α_ covariance matrix.

Afterwards, we analyzed the change in RMSD, RMSF, SASA, and PCA analysis of Mdm2 before and after modification of amino acids, as shown in Figure 3. The average RMSD value for control Mdm2 was 0.18 ± 0.03 and 0.24 ± 0.02 for modified Mdm2 (Figure 3a). RMSD results reveal that flexibility of Mdm2 increases with the modification of amino acids. Figure 3b shows that oxidized Mdm2 protein residues’ fluctuations were higher than control Mdm2, especially between 66 and 109 residues. The residues between 66 and 74 showed the highest fluctuation of ≈0.37 nm, and residues between 79 and 105 showed the highest fluctuation ≈0.55 nm. Further, we calculated the average SASA for Mdm2 before and after modified amino acids. Figure 3c shows the average SASA of 59 ± 1 and 60 ± 2 nm^2^ for control and modified Mdm2 proteins, respectively. Additionally, the magnitudes of the eigenvalues of Mdm2 control was lower than plasma-modified Mdm2; see Figure 3d. The RMSD, RMSF, and SASA values revealed that flexibility of Mdm2 increases after modification of amino acids (Leu54, Leu57, Met62, Tyr67, Val75, Val93, Phe86, and Phe91). The unbounded control Mdm2 eigenvalues magnitude obtained in our study is similar to the previously obtained value [41]. The overall flexibility of the native and oxidized structure was calculated by the trace of the diagonalized covariance matrix of the C_α_-atomic positional fluctuations. The covariance value for native and oxidized Mdm2 was 1.07 and 2.50 nm^2^, respectively. This clearly shows that overall flexibility was increased for oxidized Mdm2, which supports the RMSD, RMSF, and SASA values.

### 2.2. MD Simulation of Mdm2–p53 Complex before and after Possible CAP-Induced Modification

**Figure 4:**
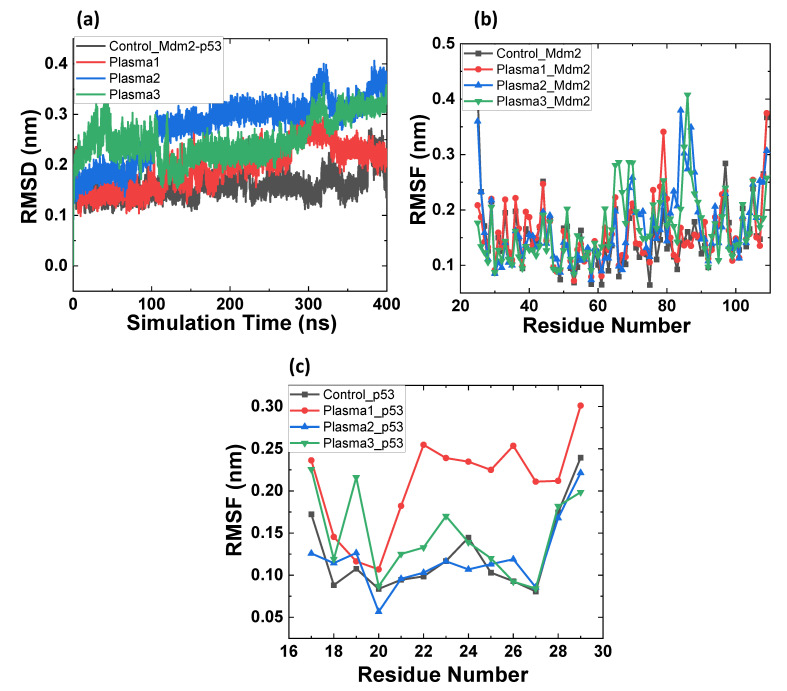
MD simulation results of the native and oxidized Mdm2–p53 protein. (**a**) Root mean square deviation (RMSD) of the C_α_ atoms, (**b**) root mean square fluctuations of Mdm2 control or oxidized in the different complex, and (**c**) root mean square fluctuations of p53 control or oxidized in the different complex.

Mdm2–p53 complex modifications were divided into 4 types: control, plasma1, plasma2, and plasma3, as discussed above. Figure 4a shows the changes in RMSD values for all the systems. The average RMSD values were 0.19 ± 0.03, 0.22 ± 0.01, 0.34 ± 0.02, and 0.32 ± 0.01 nm, for control, plasma1, plasma2, and plasma3, respectively. This shows that the control complex has the least RMSD value and plasma2 has the highest RMSD value.

To better understand the fluctuation of Mdm2 in the Mdm2–p53 complex, we calculated the RMSF values in Figure 4b. Mdm2 of plasma1 amino acids fluctuated from 31 to 44 and 76 to 80 residues, Mdm2 of plasma2 showed slight variation between 81 and 89 residues, and Mdm2 of plasma3 showed higher fluctuation from 62 to 70 and 79 to 90 residues. The highest fluctuation was shown by Mdm2 in plasma1 at residue 79 for ≈0.34 nm and in plasma3 at residue 86 for ≈0.4 nm. Further, we analyzed the RMSF values for the p53 in the Mdm2–p53 complexes in Figure 4c. RMSF value of bounded p53 revealed the highest and least fluctuation for plasma1 and plasma2, respectively.

**Figure 5:**
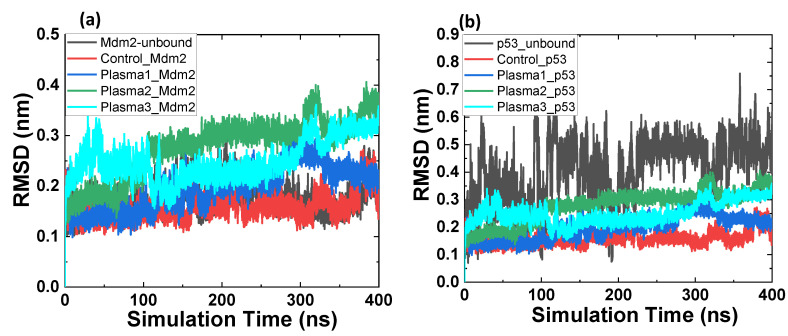
Root mean square deviation (RMSD) of the Cα atoms (**a**) Mdm2 and (**b**) p53 in Mdm2 unbounded (black), Mdm2–p53 complex, plasma1, plasma2, and plasma3.

Mdm2 amino acids’ modification gives rise to the increased flexibility of the Mdm2–p53 complex. Additionally, it was interesting to note that the RMSD value of plasma3 was less than plasma2, which means the oxidized form of Mdm2 and p53 has higher rigidness than Mdm2 (oxidized)-p53 (without oxidation). Unlike RMSD values, plasma2 shows lower fluctuation than plasma1 and plasma3. Figure 5a indicates that the RMSD of unbounded and bounded Mdm2 showed close RMSD values to each other. At the same time, RMSD values for plasma1, plasma2, and plasma3 were higher than the unbounded Mdm2 control. In contrast, p53 unbounded has higher RMSD than p53 bounded (control, plasma1, plasma2, and plasma3); see Figure 5b.

**Figure 6:**
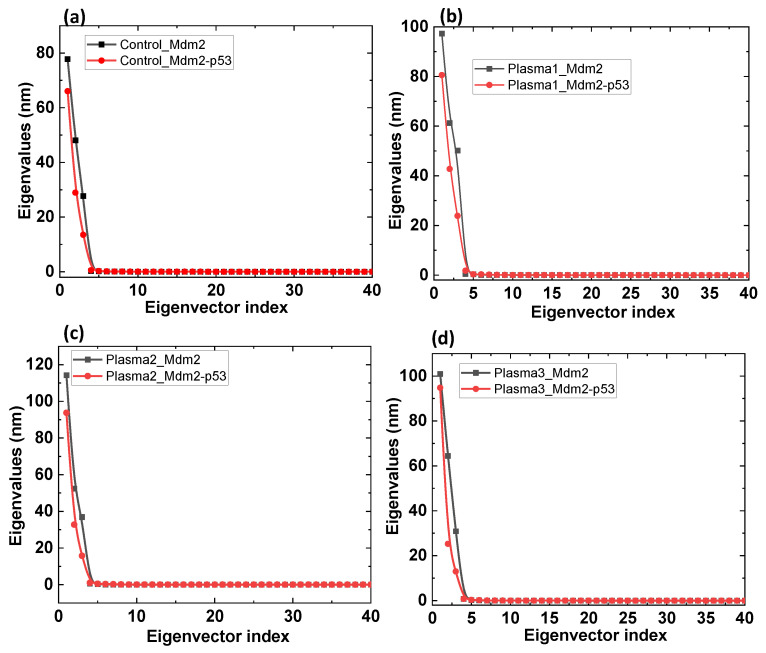
Comparison of the eigenvalues plotted against the corresponding eigenvector indices obtained from the C_α_ covariance matrix for (**a**) control Mdm2 and Mdm2–p53 complex, (**b**) plasma1 Mdm2 and Mdm2–p53 complex, (**c**) plasma2 Mdm2 and Mdm2–p53 complex, and (**d**) plasma3 Mdm2 and Mdm2–p53 complex.

Later, we plotted the eigenvalues against the corresponding eigenvector for Mdm2 and their corresponding Mdm2–p53 complex. Figure 6 shows the magnitude of the eigenvalues of Mdm2 complexed with p53, which is lower than that of the Mdm2. This indicates that the p53 binding decreased the magnitude of PC1. We observed similar behavior for all the complex systems where the oxidized or control Mdm2 have a higher eigenvalue than their corresponding complex with p53 (Figure 6). The C_α_ covariance value for Mdm2 in control complex, plasma1, plasma2, and plasma3 was 197.99, 210.12, 205.21, and 197.99 nm^2^, respectively. The C_α_ covariance value for Mdm2–p53 complex in the control complex, plasma1, plasma2, and plasma3 was 135.43, 174.30, 145.35, and 135.26 nm^2^, respectively. This clearly shows p53 binding decreases the C_α_ covariance value of Mdm2. Additionally, oxidized p53 interaction with control Mdm2 results in the highest C_α_ covariance value compared to the other systems.

**Figure 7:**
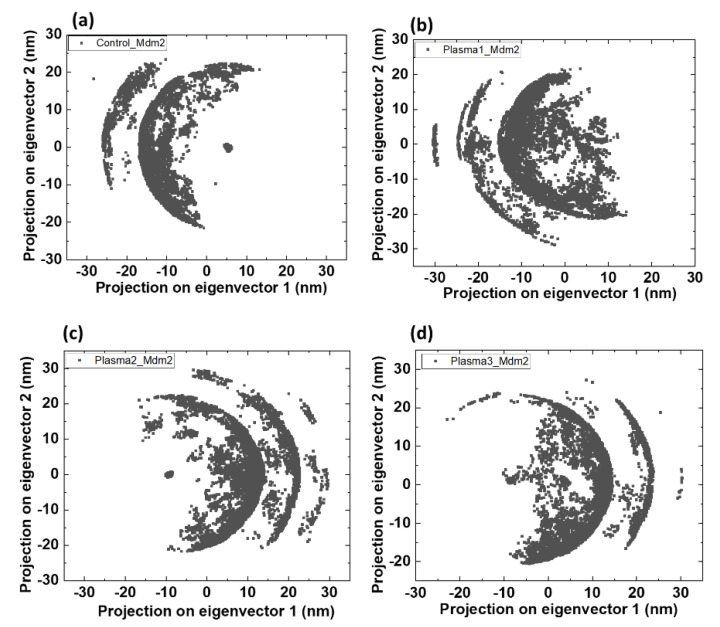
Projection of the motion of the Mdm2 protein in phase space along the first two principal eigenvectors at 300 K. (**a**) Control, (**b**) plasma1, (**c**) plasma2, and (**d**) plasma3.

Further, we calculated the total phase space before and after modifying amino acids of Mdm2 (bounded) and Mdm2–p53 complex (with and without oxidation), as shown in Figure 7, which indicates that fluctuations of the Mdm2 in different systems were confined to the first two eigenvectors. The phase spaces with Mdm2 in control, plasma1, plasma2, and plasma3 are different from each other.

**Figure 8:**
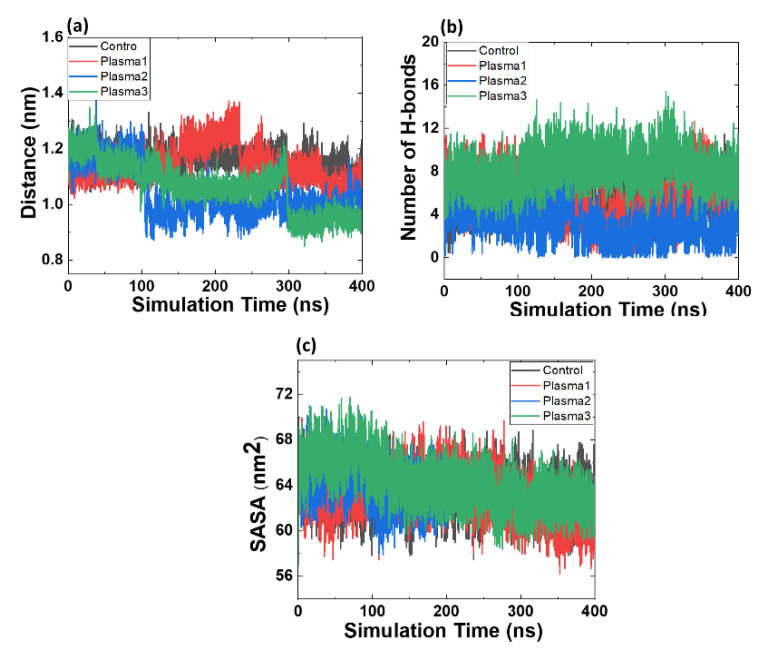
(**a**) Minimum distance formed between Mdm2 and p53 protein in the native and oxidized state, (**b**) total number of hydrogen bonds formed between Mdm2 and p53 protein in the native and oxidized state, and (**c**) solvent-accessible surface area (SASA) of Mdm2–p53 complex.

The minimum distance between the Mdm2 and p53 protein was computed for all the complexes, as shown in Figure 8a. The average distance between native Mdm2 and p53 was 1.13 ± 0.02 nm in the last 50 ns simulation period. Simultaneously, the average distance between Mdm2 and p53 in plasma1, plasma2, and plasma3 was 1.10 ± 0.03, 0.99 ± 0.04, and 0.95 ± 0.02 nm, respectively. Average distance analysis showed the slightly increased distance between Mdm2 and p53 in plasma1 than plasma2 and plasma3 complexes, which may occur due to substituting oxidized amino acid in p53 protein. However, the distance between oxidized Mdm2 and oxidized p53 was the smallest.

Hydrogen bonds (H-bonds) play an essential role in chemistry and biology. H-bonds are responsible for maintaining protein stability, and establishing the H-bonds significantly affects the protein stability or stability between the two proteins [42,43]. Figure 8b depicts the number of H-bonds formed between Mdm2–p53 protein in both native and modified states. The native complex of Mdm2–p53 protein exhibits an average of six H-bonds in the last 50 ns simulation period. The average maximum number of H-bonds bonds formed in plasma1, plasma2, and plasma3 were seven, two, and eight, respectively. Notably, the plasma2 complex has the least number of H-bonds, and plasma3 has the highest number of H-bonds, attributed to a minimum and maximum interaction between Mdm2 and p53. H-bonds also contributed to higher free energy in protein–protein interaction [24,44].

The SASA of a protein is that surface followed by the solvent molecule. Solvation effects support the rearrangement of protein structure and the protein–protein binding process. SASA was calculated for all the protein complexes of Mdm2–p53 protein. From Figure 8c, the average SASA values of native Mdm2–p53 protein were obtained. SASA for control, plasma1, plasma2, and plasma3 were 63 ± 1.6, 65 ± 1.5, 63 ± 1.2, and 64 ± 1.4 nm^2^ in the last 50 ns simulation. This revealed that SASA values slightly increase for plasma1 and plasma3 compared to native and plasma2; this indicated the oxidized amino acids of p53 influenced the SASA values.

## 3. Discussion

MD simulation results reveal that oxidized Mdm2 is more flexible than the native protein. The flexibility of the Mdm2–p53 complexes increases after oxidation. RMSD and RMSF values showed that oxidation of the Mdm2 complex results in high flexibility that might disturb the binding with p53. It was reported that overexpression of Mdm2 in human malignancies and epidermoid carcinoma inhibited cisplatin sensitivity and simultaneously downregulated p53 [1,45,46]. Overexpressed MDM2 in breast cancer cells induced resistance to doxorubicin through downregulating wtp53 [47]. Additionally, homeobox A13 protein regulated the Mdm2–p53 loop, resulting in fluorouracil (5-FU) resistance in tumor cells [48]. Overexpression of Mdm2 plays a critical factor in the tumor cells’ resistance to radiotherapy [49]. In most of the studies, the overexpression of Mdm2 reduces the p53 expression. We proposed that oxidation of Mdm2 might impact the decreased binding energies between Mdm2 and p53, resulting in the availability of p53 which results in the increased intracellular ROS in cancer cells and causes cancer cell death, as described below.

The CAP chemical composition contains many short- and long-lived reactive species such as hydroxyl radicals (^●^OH), superoxide (O_2_^●−^), nitric oxide (NO^●^), ozone (O_3_), peroxynitrite (ONOO^−^), hydroperoxyl (HO_2_^●^), hydrogen peroxide (H_2_O_2_), nitrites (NO_2_^−^), nitrates (NO_3_^−^), etc. The long-lived reactive species such as H_2_O_2_, NO_2_^−^, and NO_3_^−^ reach the cell surface and initiate redox biochemistry, resulting in cancer cell death, as described in earlier work [21]. After CAP treatment, the intracellular ROS concentration increase is generally accepted and reported in various articles [50,51,52,53,54,55,56,57]. The long-lived reactive species go through different reactions, shown below, that play an essential role in cell death [58].

NO_2_^−^ + H_2_O_2_*→* ONOO^−^ + H_2_O (1)

ONOO^−^ + H_2_O_2_ → ^1^O_2_ + H_2_O + NO_2_^−^ (2)

ONOOH → ^●^NO_2_ + ^●^OH (3)

H_2_O_2_ + ^●^OH → H_2_O + HO_2_ (4)

O_2_^●−^ + NO^●^ → ONOO^−^ (5)

H_2_O_2_ + catalase → H_2_O + 1/2 O_2_ (6)

H_2_O_2_ and NO_2_^−^ react to produce ONOO^−^ (Equation (1)). ONOO^−^ reacts with H_2_O_2_ to generate ^1^O_2_ and NO_2_^−^ (Equation (2)). ONOOH decomposes to ^●^NO_2_ and ^●^OH (Equation (3)), and ^●^OH induces lipid peroxidation [59]. Another possibility is the reaction of ^●^OH with H_2_O_2_ to form HO_2_^●^ (Equation (4)).

The O_2_^●−^ produced from the NOX enzyme reacts with HO_2_^●^ to generate the ^1^O_2_. We have not included this reaction in our system because we recently reported that NOX1 expression decreases after plasma treatment [23]. It is highly possible that NOX1 will not generate the O_2_^●−^ or generate a lesser amount. Therefore, there was not enough ^1^O_2_ to completely deactivate the catalase. These results are supported by recently published work by Bengtson and Bogaerts [60]. The authors developed a mathematical model and proposed that catalase-dependent apoptotic pathways did not contribute to the CAP-induced anticancer activity [60]. Although, in the present study, we proposed that the oxidized Mdm2 does not inhibit p53, which might lead to increases in the p53 availability, deactivating the catalase through p53-inducible genes (PIG genes). It was already known that p53 promotes oxidative damage by regulating its transcriptional targets such as PIG genes [61]; PIG3 genes bind to catalase and inhibit its activity [62]. A recent review by Kim et al. indicated the important role of p53 in radiation-induced oxidative stress [63]. Reports stated the increased intracellular ROS level activated the p53 through JNK signaling, which was accountable for pro-oxidant gene upregulation [64,65,66]. Additionally, p53 suppresses the antioxidants associated with the nuclear-factor-E2-related factor (Nrf2) [67]. Hence, we assumed that oxidized Mdm2 does not inhibit p53 protein in CAP-treated cancer cells, which results in cancer cell death through oxidative stress.

## 4. Materials and Methods

### Molecular Dynamics (MD) Simulations

We have taken the molecular structure of Mdm2–p53 protein PDB IDs 1YCR for the MD simulations. We used UCSF Chimera v.1.13.1. (Molecular graphics and analyses performed with UCSF Chimera, developed by the Resource for Biocomputing, Visualization, and Informatics at the University of California, USA, with support from NIH P41-GM103311) to separate the structure of Mdm2 and p53 from Mdm2–p53 complex [68]. MD simulations were performed on Mdm2, p53, and Mdm2–p53 complex using GROMACS 5.1.2 package by research teams in the University of Groningen and Uppsala University [69] using the GROMOS54A7 force field [70]. The protein was solvated with water, described by the simple point charge (SPC) explicit solvent model [71]. Later, the system was neutralized by Na^+^ or Cl^−^ ions replacing the water molecules. The length of the simulation box was set as 6.2 × 6.2 × 6.2 nm^3^, which contained 7500 water molecules. The system was energy minimized with the steepest descent method. The NPT equilibration was first carried out for 10 ns at 300 K and 1 bar by applying positional restraints (force constant of 1000 kJ mol^−1^ nm^−2^) on the protein’s heavy atoms. This was done to keep the system as close as possible to its crystal structure. We employed the stochastic velocity rescaling thermostat with a time constant of 0.1 ps [72], Parrinello–Rahman barostat with a time constant of 2 ps [73], and isothermal compressibility of 4.5 × 10^−5^ bar^−1^ [74]. The cut-off radii of the van der Waals and Coulomb interaction were 1 nm.

Additionally, the long-range electrostatic interaction was calculated by the PME (particle mesh Ewald summation) [75] and applying long-range dispersion corrections for both energy and pressure. After the equilibration, we performed the unrestrained (normal) MD simulations for 400 ns, of which the last 50 ns were used to calculate the average values. In all simulations, a time step of 2 fs was used. Finally, we calculated the root mean square deviation (RMSD) of the C_α_ atoms, solvent-accessible surface area (SASA), root mean square fluctuations (RMSF), total number of H-bonds between Mdm2 and p53, minimum distance formed between Mdm2 and p53, principal component analysis (PCA), etc. Besides simulating the native Mdm2, p53, and Mdm2–p53 complex structures in water, we also performed the same simulations to an oxidized form of Mdm2, p53, and Mdm2–p53 complex to better understand the effect of oxidized amino acids on the protein. The Mdm2 and p53 oxidized amino acids were based on the most probable plasma oxidized products [38]. We obtained the GROMOS54A7 force field parameters for the oxidized Mdm2 and p53 from references [39,40].

## 5. Conclusions

Our MD study showed that the flexibility increased for the complexes with oxidized Mdm2 or oxidized p53 compared to non-oxidized protein complex. RMSD, RMSF, and SASA values revealed that flexibility increases for unbounded oxidized Mdm2 as compared to non-oxidized Mdm2. On the other hand, no significant change was observed in unbounded p53 before and after oxidation. The least and highest RMSD values were obtained for the control Mdm2–p53 complex and plasma2, respectively. This reveals that the flexibility of the Mdm2–p53 complex increases with oxidized Mdm2 as compared to the control complex. Additionally, bounded Mdm2 showed the highest RMSF value in plasma2 and plasma3, while bounded p53 revealed the highest fluctuation in plasma1. This further supports that fluctuation increases more for oxidized Mdm2 complex than non-oxidized Mdm2 complex. Therefore, we proposed that plasma-induced oxidized Mdm2 does not inhibit p53, which might be the possible reason for catalase deactivation by p53 rather than singlet oxygen generated in plasma-induced reactions, which results in increased intracellular ROS in the plasma-treated cancer cells. However, more studies are required to prove our assumption for p53-induced cancer cell death in plasma oncology.

## Data Availability

The authors have all the data.

## References

[1] Hou H., Sun D., Zhang X. (2019). The role of MDM2 amplification and overexpression in therapeutic resistance of malignant tumors. Cancer Cell Int..

[2] Picksley S.M., Lane D.P. (1993). What the papers say: The p53-mdm2 autoregulatory feedback loop: A paradigm for the regulation of growth control by p53?. BioEssays.

[3] Levine A.J. (1997). p53, the Cellular Gatekeeper for Growth and Division. Cell.

[4] Vassilev L.T., Vu B.T., Graves B., Carvajal D., Podlaski F., Filipovic Z., Kong N., Kammlott U., Lukacs C., Klein C. (2004). In Vivo Activation of the p53 Pathway by Small-Molecule Antagonists of MDM2. Science.

[5] Wasylyk C., Salvi R., Argentini M., Dureuil C., Delumeau I., Abecassis J., Debussche L., Wasylyk B. (1999). p53 mediated death of cells overexpressing MDM2 by an inhibitor of MDM2 interaction with p53. Oncogene.

[6] Chène P. (2004). Inhibition of the p53-MDM2 Interaction: Targeting a Protein-Protein Interface. Mol. Cancer Res..

[7] Verma S., Grover S., Tyagi C., Goyal S., Jamal S., Singh A., Grover A. (2016). Hydrophobic interactions are a key to MDM2 inhibition by polyphenols as revealed by molecular dynamics simulations and MM/PBSA free energy calculations. PLoS ONE.

[8] Zhao J., Wang M., Chen J., Luo A., Wang X., Wu M., Yin D., Liu Z. (2002). The initial evaluation of non-peptidic small-molecule HDM2 inhibitors based on p53–HDM2 complex structure. Cancer Lett..

[9] Stoll R., Renner C., Hansen S., Palme S., Klein C., Belling A., Zeslawski W., Kamionka M., Rehm T., Mühlhahn P. (2001). Chalcone Derivatives Antagonize Interactions between the Human Oncoprotein MDM2 and p53 †. Biochemistry.

[10] Bautista A.D., Appelbaum J.S., Craig C.J., Michel J., Schepartz A. (2010). Bridged β 3-Peptide Inhibitors of p53-hDM2 Complexation: Correlation between Affinity and Cell Permeability. J. Am. Chem. Soc..

[11] Sahin I., Zhang S., Navaraj A., Zhou L., Dizon D., Safran H., El-Deiry W.S. (2020). AMG-232 sensitizes high MDM2-expressing tumor cells to T-cell-mediated killing. Cell Death Discov..

[12] Adil B.H., Al-Shammari A.M., Murbat H.H. (2020). Breast cancer treatment using cold atmospheric plasma generated by the FE-DBD scheme. Clin. Plasma Med..

[13] Irani S., Shahmirani Z., Atyabi S.M., Mirpoor S. (2015). Induction of growth arrest in colorectal cancer cells by cold plasma and gold nanoparticles. Arch. Med. Sci..

[14] Vaquero J., Judée F., Vallette M., Decauchy H., Arbelaiz A., Aoudjehane L., Scatton O., Gonzalez-Sanchez E., Merabtene F., Augustin J. (2020). Cold-atmospheric plasma induces tumor cell death in preclinical in vivo and in vitro models of human cholangiocarcinoma. Cancers.

[15] Karki S.B., Gupta T.T., Yildirim-Ayan E., Eisenmann K.M., Ayan H. (2017). Investigation of non-thermal plasma effects on lung cancer cells within 3D collagen matrices. J. Phys. D Appl. Phys..

[16] Ishaq M., Han Z.J., Kumar S., Evans M.D.M., Ostrikov K.K. (2015). Atmospheric-Pressure Plasma- and TRAIL-Induced Apoptosis in TRAIL-Resistant Colorectal Cancer Cells. Plasma Process. Polym..

[17] Köritzer J., Boxhammer V., Schäfer A., Shimizu T., Klämpfl T.G., Li Y.-F., Welz C., Schwenk-Zieger S., Morfill G.E., Zimmermann J.L. (2013). Restoration of Sensitivity in Chemo—Resistant Glioma Cells by Cold Atmospheric Plasma. PLoS ONE.

[18] Lee S., Lee H., Jeong D., Ham J., Park S., Choi E.H., Kim S.J. (2017). Cold atmospheric plasma restores tamoxifen sensitivity in resistant MCF-7 breast cancer cell. Free Radic. Biol. Med..

[19] Yang H., Lu R., Xian Y., Gan L., Lu X., Yang X. (2015). Effects of atmospheric pressure cold plasma on human hepatocarcinoma cell and its 5-fluorouracil resistant cell line. Phys. Plasmas.

[20] Park S., Kim H., Ji H.W., Kim H.W., Yun S.H., Choi E.H., Kim S.J. (2019). Cold atmospheric plasma restores paclitaxel sensitivity to paclitaxel-resistant breast cancer cells by reversing expression of resistance-related genes. Cancers.

[21] Song C.-H., Attri P., Ku S.-K., Han I., Bogaerts A., Choi E.H. (2021). Cocktail of reactive species generated by cold atmospheric plasma: Oral administration induces non-small cell lung cancer cell death. J. Phys. D Appl. Phys..

[22] Bauer G. (2018). Targeting Protective Catalase of Tumor Cells with Cold Atmospheric Plasma-Activated Medium (PAM). Anticancer Agents Med. Chem..

[23] Attri P., Park J.H., De Backer J., Kim M., Yun J.H., Heo Y., Dewilde S., Shiratani M., Choi E.H., Lee W. (2020). Structural modification of NADPH oxidase activator (Noxa 1) by oxidative stress: An experimental and computational study. Int. J. Biol. Macromol..

[24] Attri P., Koga K., Shiratani M. (2020). Possible impact of plasma oxidation on the structure of C-terminal domain of SARS-CoV-2 spike protein: A computational study. Appl. Phys. Express.

[25] Attri P., Razzokov J., Yusupov M., Koga K., Shiratani M., Bogaerts A. (2020). Influence of osmolytes and ionic liquids on the Bacteriorhodopsin structure in the absence and presence of oxidative stress: A combined experimental and computational study. Int. J. Biol. Macromol..

[26] Ke Z., Huang Q. (2013). Inactivation and Heme Degradation of Horseradish Peroxidase Induced by Discharge Plasma. Plasma Process. Polym..

[27] Attri P., Han J., Choi S., Choi E.H., Bogaerts A., Lee W. (2018). CAP modifies the structure of a model protein from thermophilic bacteria: Mechanisms of CAP-mediated inactivation. Sci. Rep..

[28] Takai E., Kitano K., Kuwabara J., Shiraki K. (2012). Protein Inactivation by Low-temperature Atmospheric Pressure Plasma in Aqueous Solution. Plasma Process. Polym..

[29] Choi S., Attri P., Lee I., Oh J., Yun J.-H., Park J.H., Choi E.H., Lee W. (2017). Structural and functional analysis of lysozyme after treatment with dielectric barrier discharge plasma and atmospheric pressure plasma jet. Sci. Rep..

[30] Attri P., Kim M., Sarinont T., Ha Choi E., Seo H., Cho A.E., Koga K., Shiratani M. (2017). The protective action of osmolytes on the deleterious effects of gamma rays and atmospheric pressure plasma on protein conformational changes. Sci. Rep..

[31] Attri P., Kumar N., Park J.H., Yadav D.K., Choi S., Uhm H.S., Kim I.T., Choi E.H., Lee W. (2015). Influence of reactive species on the modification of biomolecules generated from the soft plasma. Sci. Rep..

[32] Li H.-P., Wang L.-Y., Li G., Jin L.-H., Le P.-S., Zhao H.-X., Xing X.-H., Bao C.-Y. (2011). Manipulation of Lipase Activity by the Helium Radio-Frequency, Atmospheric-Pressure Glow Discharge Plasma Jet. Plasma Process. Polym..

[33] Attri P., Sarinont T., Kim M., Amano T., Koga K., Cho A.E., Choi E.H., Shiratani M. (2015). Influence of ionic liquid and ionic salt on protein against the reactive species generated using dielectric barrier discharge plasma. Sci. Rep..

[34] Attri P., Choi E.H. (2013). Influence of Reactive Oxygen Species on the Enzyme Stability and Activity in the Presence of Ionic Liquids. PLoS ONE.

[35] Zhang H., Xu Z., Shen J., Li X., Ding L., Ma J., Lan Y., Xia W., Cheng C., Sun Q. (2015). Effects and Mechanism of Atmospheric-Pressure Dielectric Barrier Discharge Cold Plasmaon Lactate Dehydrogenase (LDH) Enzyme. Sci. Rep..

[36] Zou R., Zhou Y., Wang Y., Kuang G., Ågren H., Wu J., Tu Y. (2020). Free Energy Profile and Kinetics of Coupled Folding and Binding of the Intrinsically Disordered Protein p53 with MDM2. J. Chem. Inf. Model..

[37] Zwier M.C., Pratt A.J., Adelman J.L., Kaus J.W., Zuckerman D.M., Chong L.T. (2016). Efficient Atomistic Simulation of Pathways and Calculation of Rate Constants for a Protein-Peptide Binding Process: Application to the MDM2 Protein and an Intrinsically Disordered p53 Peptide. J. Phys. Chem. Lett..

[38] Takai E., Kitamura T., Kuwabara J., Ikawa S., Yoshizawa S., Shiraki K., Kawasaki H., Arakawa R., Kitano K. (2014). Chemical modification of amino acids by atmospheric-pressure cold plasma in aqueous solution. J. Phys. D Appl. Phys..

[39] Margreitter C., Petrov D., Zagrovic B. (2013). Vienna-PTM web server: A toolkit for MD simulations of protein post-translational modifications. Nucleic Acids Res..

[40] Margreitter C., Reif M.M., Oostenbrink C. (2017). Update on phosphate and charged post-translationally modified amino acid parameters in the GROMOS force field. J. Comput. Chem..

[41] Cheng W., Liang Z., Wang W., Yi C., Li H., Zhang S., Zhang Q. (2016). Insight into binding modes of p53 and inhibitors to MDM2 based on molecular dynamic simulations and principal component analysis. Mol. Phys..

[42] Gerlt J.A., Kreevoy M.M., Cleland W.W., Frey P.A. (1997). Understanding enzymic catalysis: The importance of short, strong hydrogen bonds. Chem. Biol..

[43] George Priya Doss C., Nagasundaram N. (2014). Molecular Docking and Molecular Dynamics Study on the Effect of ERCC1 Deleterious Polymorphisms in ERCC1-XPF Heterodimer. Appl. Biochem. Biotechnol..

[44] Erijman A., Rosenthal E., Shifman J.M. (2014). How structure defines affinity in protein-protein interactions. PLoS ONE.

[45] Kondo S., Barnett G.H., Hara H., Morimura T., Takeuchi J. (1995). MDM2 protein confers the resistance of a human glioblastoma cell line to cisplatin-induced apoptosis. Oncogene.

[46] Hayashi S., Ozaki T., Yoshida K., Hosoda M., Todo S., Akiyama S., Nakagawara A. (2006). p73 and MDM2 confer the resistance of epidermoid carcinoma to cisplatin by blocking p53. Biochem. Biophys. Res. Commun..

[47] Suzuki A., Toi M., Yamamoto Y., Saji S., Muta M., Tominaga T. (1998). Role of MDM2 overexpression in doxorubicin resistance of breast carcinoma. Jpn. J. Cancer Res..

[48] Han Y., Song C., Wang J., Tang H., Peng Z., Lu S. (2018). HOXA13 contributes to gastric carcinogenesis through DHRS2 interacting with MDM2 and confers 5-FU resistance by a p53-dependent pathway. Mol. Carcinog..

[49] Koom W.S., Park S.Y., Kim W., Kim M., Kim J.S., Kim H., Choi I.K., Yun C.O., Seong J. (2012). Combination of radiotherapy and adenovirus-mediated p53 gene therapy for MDM2-overexpressing hepatocellular carcinoma. J. Radiat. Res..

[50] Kurita H., Minamijima Y., Takashima K. (2020). Characterization of intracellular reactive species production stimulated by cold atmospheric pressure plasma irradiation. Int. J. Plasma Environ. Sci. Technol..

[51] Kumar N., Attri P., Dewilde S., Bogaerts A. (2018). Inactivation of human pancreatic ductal adenocarcinoma with atmospheric plasma treated media and water: A comparative study. J. Phys. D Appl. Phys..

[52] Mateu-Sanz M., Tornín J., Brulin B., Khlyustova A., Ginebra M.P., Layrolle P., Canal C. (2020). Cold plasma-treated ringer’s saline: A weapon to target osteosarcoma. Cancers.

[53] Xu D., Wang B., Xu Y., Chen Z., Cui Q., Yang Y., Chen H., Kong M.G. (2016). Intracellular ROS mediates gas plasma-facilitated cellular transfection in 2D and 3D cultures. Sci. Rep..

[54] Turrini E., Laurita R., Stancampiano A., Catanzaro E., Calcabrini C., Maffei F., Gherardi M., Colombo V., Fimognari C. (2017). Cold Atmospheric Plasma Induces Apoptosis and Oxidative Stress Pathway Regulation in T-Lymphoblastoid Leukemia Cells. Oxid Med. Cell. Longev..

[55] Kumar N., Shaw P., Uhm H.S., Choi E.H., Attri P. (2017). Influence of Nitric Oxide generated through microwave plasma on L6 skeletal muscle cell myogenesis via oxidative signaling pathways. Sci. Rep..

[56] Bekeschus S., Lippert M., Diepold K., Chiosis G., Seufferlein T., Azoitei N. (2019). Physical plasma-triggered ROS induces tumor cell death upon cleavage of HSP90 chaperone. Sci. Rep..

[57] Moniruzzaman R., Rehman M.U., Zhao Q.-L., Jawaid P., Mitsuhashi Y., Imaue S., Fujiwara K., Ogawa R., Tomihara K., Saitoh J. (2018). Roles of intracellular and extracellular ROS formation in apoptosis induced by cold atmospheric helium plasma and X-irradiation in the presence of sulfasalazine. Free Radic. Biol. Med..

[58] Bauer G. (2015). Increasing the endogenous NO level causes catalase inactivation and reactivation of intercellular apoptosis signaling specifically in tumor cells. Redox Biol..

[59] Tejero I., González-Lafont À., Lluch J.M., Eriksson L.A. (2007). Theoretical Modeling of Hydroxyl-Radical-Induced Lipid Peroxidation Reactions. J. Phys. Chem. B.

[60] Bengtson C., Bogaerts A. (2020). On the Anti-Cancer Effect of Cold Atmospheric Plasma and the Possible Role of Catalase-Dependent Apoptotic Pathways. Cells.

[61] Liu X., Fan L., Lu C., Yin S., Hu H. (2020). Functional Role of p53 in the Regulation of Chemical-Induced Oxidative Stress. Oxid. Med. Cell. Longev..

[62] Kang M.Y., Kim H.B., Piao C., Lee K.H., Hyun J.W., Chang I.Y., You H.J. (2013). The critical role of catalase in prooxidant and antioxidant function of p53. Cell Death Differ..

[63] Kim K., Lee S., Seo D., Kang J., Seong K.M., Youn H. (2019). Cellular Stress Responses in Radiotherapy. Cells.

[64] Liu B., Bhatt D., Oltvai Z.N., Greenberger J.S., Bahar I. (2015). Significance of p53 dynamics in regulating apoptosis in response to ionizing radiation and polypharmacological strategies. Sci. Rep..

[65] Shi Y., Nikulenkov F., Zawacka-Pankau J., Li H., Gabdoulline R., Xu J., Eriksson S., Hedström E., Issaeva N., Kel A. (2014). ROS-dependent activation of JNK converts p53 into an efficient inhibitor of oncogenes leading to robust apoptosis. Cell Death Differ..

[66] Italiano D., Lena A.M., Melino G., Candi E. (2012). Identification of NCF2/p67phox as a novel p53 target gene. Cell Cycle.

[67] Faraonio R., Vergara P., Di Marzo D., Pierantoni M.G., Napolitano M., Russo T., Cimino F. (2006). p53 Suppresses the Nrf2-dependent Transcription of Antioxidant Response Genes. J. Biol. Chem..

[68] Pettersen E.F., Goddard T.D., Huang C.C., Couch G.S., Greenblatt D.M., Meng E.C., Ferrin T.E. (2004). UCSF Chimera?A visualization system for exploratory research and analysis. J. Comput. Chem..

[69] Abraham M.J., Murtola T., Schulz R., Páll S., Smith J.C., Hess B., Lindahl E. (2015). GROMACS: High Performance Molecular Simulations through Multi-Level Parallelism from Laptops to Supercomputers. SoftwareX.

[70] Schmid N., Eichenberger A.P., Choutko A., Riniker S., Winger M., Mark A.E., van Gunsteren W.F. (2011). Definition and testing of the GROMOS force-field versions 54A7 and 54B7. Eur. Biophys. J..

[71] Berendsen H.J.C., Postma J.P.M., van Gunsteren W.F., Hermans J., Pullman B. (1981). Interaction Models for Water in Relation to Protein Hydration. Intermolecular Forces. The Jerusalem Symposia on Quantum Chemistry and Biochemistry.

[72] Bussi G., Donadio D., Parrinello M. (2007). Canonical sampling through velocity rescaling. J. Chem. Phys..

[73] Parrinello M., Rahman A. (1981). Polymorphic transitions in single crystals: A new molecular dynamics method. J. Appl. Phys..

[74] Berendsen H.J.C., Postma J.P.M., van Gunsteren W.F., DiNola A., Haak J.R. (1984). Molecular dynamics with coupling to an external bath. J. Chem. Phys..

[75] Darden T., York D., Pedersen L. (1993). Particle mesh Ewald: An N log( N ) method for Ewald sums in large systems. J. Chem. Phys..

